# Synthesis of π-conjugated polycyclic compounds by late-stage extrusion of chalcogen fragments

**DOI:** 10.3762/bjoc.20.30

**Published:** 2024-02-15

**Authors:** Aissam Okba, Pablo Simón Marqués, Kyohei Matsuo, Naoki Aratani, Hiroko Yamada, Gwénaël Rapenne, Claire Kammerer

**Affiliations:** 1 CEMES, Université de Toulouse, CNRS, 29 rue Marvig, F-31055 Toulouse Cedex 4, France; 2 Division of Materials Science, Nara Institute of Science and Technology, NAIST, 8916-5 Takayama-cho, Ikoma, Nara 630-0192, Japan; 3 Institute for Chemical Research, Kyoto University, Gokasho, Uji, Kyoto 611-0011, Japan

**Keywords:** arenes, chalcogens, extrusion, fused-ring systems, precursor approach

## Abstract

The “*precursor approach*” has proved particularly valuable for the preparation of insoluble and unstable π-conjugated polycyclic compounds (π-CPCs), which cannot be synthesized via in-solution organic chemistry, for their improved processing, as well as for their electronic investigation both at the material and single-molecule scales. This method relies on the synthesis and processing of soluble and stable direct precursors of the target π-CPCs, followed by their final conversion in situ, triggered by thermal activation, photoirradiation or redox control. Beside well-established reactions involving the elimination of carbon-based small molecules, i.e., retro-Diels–Alder and decarbonylation processes, the late-stage extrusion of chalcogen fragments has emerged as a highly promising synthetic tool to access a wider variety of π-conjugated polycyclic structures and thus to expand the potentialities of the “*precursor approach*” for further improvements of molecular materials’ performances. This review gives an overview of synthetic strategies towards π-CPCs involving the ultimate elimination of chalcogen fragments upon thermal activation, photoirradiation and electron exchange.

## Introduction

π-Conjugated polycyclic compounds (π-CPCs), including polycyclic aromatic hydrocarbons and their heteroatom-based counterparts, have attracted the attention of the scientific community for two centuries already. Initially, major interest resulted from their structural, optical and electronic properties at the molecular level, but the last decades witnessed the rise of molecular materials and π-conjugated polycyclic compounds appeared as particularly valuable in this field. Indeed, many of them demonstrated excellent performances as active components of electronic devices such as organic field effect transistors (OFETs), organic photovoltaic cells (OPVs) and organic light-emitting diodes (OLEDs). This has prompted an ever-increasing number of research groups to design new synthetic routes towards π-CPCs, aiming at i) new organic structures for improved performances, ii) higher synthetic efficiency and modularity, and iii) better processability of target compounds to build opto-electronic devices [1–8]. In this regard, the planar character of most (non-substituted) π-CPCs represents a challenge, as it results in very low solubility in common organic solvents due to favorable intermolecular π–π stacking interactions. This inherently hampers the purification of the target compounds, thus limiting their final purity, and restricts the processing techniques to physical vapor deposition. In addition, some π-CPCs appear to be sensitive to oxidative and/or photolytic degradation, which hinders their use in devices under ambient conditions.

**Scheme 1:**
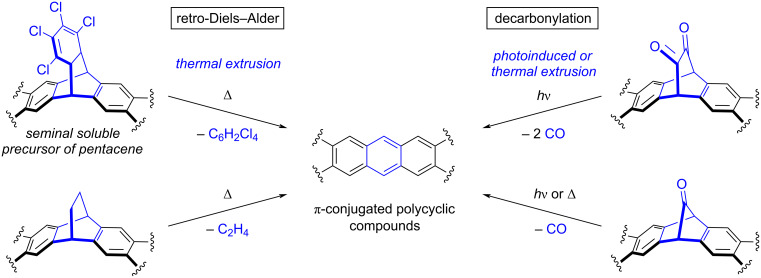
“*Precursor approach*” for the synthesis of π-conjugated polycyclic compounds, with the thermally- or photoinduced late-stage extrusion of carbon-based fragments resulting from retro-Diels–Alder and decarbonylation reactions. The seminal design of pentacene’s soluble precursor, as reported by Müllen and Herwig [14], is depicted in the top left.

The case of acenes is representative of this situation. Acenes are a class of aromatic compounds composed of linearly fused benzene rings, which can be regarded as the narrowest graphene nanoribbons. They are highly promising p-type organic semiconductors, but suffer from insolubility and instability leading to dimerization and/or endoperoxide formation. To overcome these issues, the “*precursor approach*” was developed and relies on the synthesis, purification and characterization of soluble and stable precursors of the target active π-CPCs, followed by their (solution) processing and their final deprotection once assembled in thin-films or crystals, or adsorbed on metallic substrates [9–12]. Conversion of the non-planar soluble precursor into the flat π-conjugated target compound is triggered on demand by a stimulus such as thermal activation, irradiation with light or injection of electrons, leading to the elimination of small volatile molecules. Importantly, this ultimate synthetic step, performed in situ, should be quantitative and should not require the addition of chemical reagents, since any non-volatile byproduct would be trapped in the material and thus impact its performances. The “*precursor approach*” was introduced by Herwig and Müllen as early as in 1996 for pentacene, which was ultimately obtained in a thin-film by a thermally-activated retro-Diels–Alder reaction from a tailor-made tetrachlorobenzene-pentacene soluble adduct (Scheme 1, top left) [13–14]. Over the years, thermally-induced retrocycloadditions were further exploited from bridged bicyclo[2.2.2]octadiene solubilizing fragments to generate (hetero)acenes and larger bidimensional π-CPCs such as extended porphyrin derivatives with the concomitant release of ethylene (Scheme 1, bottom left) [9,11,15]. It is important to note here that in the “*precursor approach*”, a synthetic strategy has to be devised according to a multi-step route to prepare the soluble precursor, in turn specifically designed to deliver the target π-CPC after activation in situ. This is thus fundamentally different from the approach consisting in reacting a pristine acene with a dienophile to transiently form a cycloadduct with increased solubility for processing purposes, and unmasking it afterwards via a retro-Diels–Alder reaction [16–19].

In parallel to retro-Diels–Alder reactions, another efficient strategy for the in situ aromatization of target π-CPCs relies on the extrusion of carbon monoxide (CO) from soluble precursors incorporating bridging ketone fragment(s). In contrast with the thermal activation required in retro-Diels–Alder reactions, photoirradiation of α-diketones triggers a Strating–Zwanenburg decarbonylation leading to the release of two CO molecules (Scheme 1, top right). After a proof of concept on pentacene with soluble precursors including a bicyclo[2.2.2]octane-2,3-dione framework [20–21], α-diketones were intensely exploited as soluble photoprecursors for the synthesis of acenes of increasing length up to undecacene, in matrices [22–25] or in a single crystal [26]. This strategy has also been extended to the synthesis of dyads involving an acene moiety combined with a chromophore [27]. Alternatively, when inserting a monoketone bridging fragment, i.e., a norbornadien-7-one moiety, in the soluble precursor, recovery of the target π-CPC is possible via a cheletropic decarbonylation step upon photolysis or thermolysis (Scheme 1, bottom right). This dual activation mode has led to the solid-state formation, on a preparative scale, of higher acenes up to nonacene [28–33] and bidimensional structures such as starphenes [34] from custom-made stable and soluble precursors. Interestingly, extrusion of CO from mono- or α-diketone precursors has also been achieved on metallic surfaces under ultra-high vacuum (UHV) conditions upon thermal annealing, photoirradiation or activation by the tip of a scanning tunneling microscope (STM) [11,34–36]. Owing to the subatomic resolution now available with scanning probe microscopy techniques (STM and non-contact atomic force microscopy, nc-AFM), the “*precursor approach*” has opened the way for in-depth structural and electronic investigations at the single molecule scale of π-CPCs, which could otherwise not be synthesized and/or deposited on surface.

As seen above, the “*precursor approach*” has proved particularly valuable i) for the preparation of insoluble and unstable π-CPCs which cannot be synthesized (and purified) via conventional in-solution organic chemistry techniques, ii) for their improved processing, as well as iii) for their in situ structural characterization and electronic investigation both at the material and single-molecule scales. Reactions involving the elimination of carbon-based small molecules, i.e., retro-Diels–Alder and decarbonylation processes, have been predominantly exploited as an ultimate step performed in situ to release the target compounds. However, the bridging character of the bicyclo[2.2.2]octadiene, bicyclo[2.2.2]octane-2,3-dione and norbornadien-7-one fragments in the appropriate soluble precursors inherently limits the applicability of these reactions to the synthesis of π-CPCs containing linearly-fused benzene rings in their core scaffold (i.e., at least an anthracene pattern), or to the deprotection of peripheral benzene rings. To reach a wider variety of structures, novel synthetic strategies in line with the “*precursor approach*” have been recently reported, in which chalcogen-containing precursors undergo a ring contraction combined with the extrusion of a chalcogen fragment in the ultimate step. In view of the diversity of π-conjugated polycyclic structures potentially accessible via this route, the extrusion of chalcogen fragments appears as a highly promising synthetic tool to complement the retro-Diels–Alder and decarbonylation reactions mostly exploited up to now, and thus to expand the potentialities of the “*precursor approach*” for further improvements of molecular materials performances.

Since the synthesis of π-CPCs involving as final step the elimination of C-based molecules, i.e., a retro-Diels–Alder reaction or a decarbonylation, has already been thoroughly reviewed in the past years [4–5,9–12], this review focuses on the late-stage extrusion of chalcogen fragments triggered by thermal activation, photoirradiation and electron exchange as a powerful synthetic strategy to prepare a variety of π-CPCs.

## Review

### Contraction of 7-membered rings: chalcogen extrusion from heteropines

**Scheme 2:**
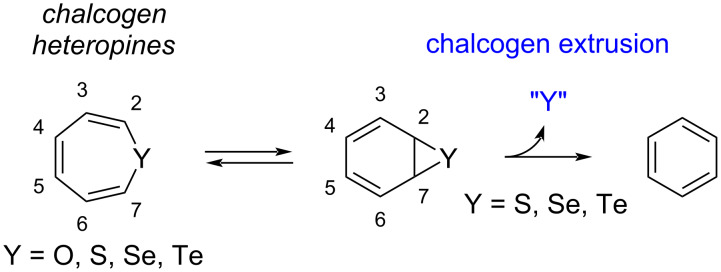
Valence isomerization of chalcogen heteropines and subsequent cheletropic extrusion in the case of sulfur, selenium and tellurium derivatives.

The family of chalcogen heteropines, i.e., cycloheptatriene analogues incorporating one group-16 atom, have attracted attention from the chemists’ community since the 1950s, when the first syntheses of oxepine [37] and thiepine [38] were reported (Scheme 2). Analogues containing heavier atoms such as Se [39] and Te [40] appeared later in the literature. In relationship with its structure, this class of compounds is prone to valence isomerization via 6π-electrocyclization leading to the formation of heteroatom-containing norcaradiene, and oxepine has been shown to exist in equilibrium with its valence isomer benzene oxide [37]. In contrast with the parent oxepine, which is isolable at room temperature, other group-16 heteropines suffer from thermal instability. Indeed, the low activation barrier for S, Se or Te cheletropic extrusion from the norcaradiene valence isomer, combined with the irreversibility of this process, shifts the tautomerization equilibrium and delivers benzene as the organic product along with inorganic species [41–44]. Over the years, many efforts were thus devoted to the synthesis of thiepine derivatives with increased thermal stability, with the aim to disfavor the formation of the transient thianorcaradiene intermediate as a way to prevent the sulfur extrusion [44]. It appeared that thiepines carrying sterically demanding substituents on the sulfur α-positions as well as annelated analogues show substantially increased lifetimes, owing to steric and electronic effects [45]. With these criteria in mind, a variety of thermally-stable annelated thiepines were progressively isolated as a result of very diverse synthetic strategies [46–49], which were further applied to the preparation of stable selenepines and tellurepines [50–53].

**Scheme 3:**
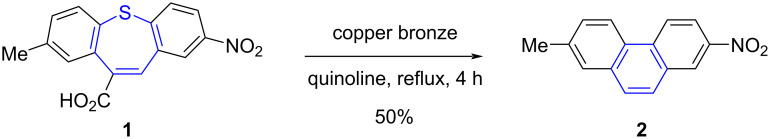
Early example of phenanthrene synthesis via a chemically-induced S-extrusion (and concomitant decarboxylation) from a dibenzothiepine precursor [56].

Extrusion reactions from sulfur-, selenium- and tellurium-containing compounds have long been recognized as valuable synthetic tools in organic chemistry [54], with the most famous S-extrusion process to generate new C–C bonds probably being the Ramberg–Bäcklund reaction [55]. In the case of heteropines, chalcogen extrusion leads to a six-membered ring via the intramolecular formation of a C–C bond, and this reactivity pattern was exploited as early as in the 1950s to generate in a controlled way polycyclic aromatic compounds from isolable heteropine precursors. By way of example, the phenanthrene derivative **2** was obtained by heating the dibenzo[*b*,*f*]thiepine precursor **1** in the presence of copper bronze in refluxing quinoline, thus triggering a S-extrusion along with a decarboxylation reaction (Scheme 3) [56]. From a retrosynthetic point of view, this example nicely illustrates the fact that polyannelated thiepines, and more generally S-, Se- and Te-based heteropines, are straightforward precursors of π-CPCs bearing non linearly-fused benzene rings. In addition, the non-planar geometry of such heteropines appears as an asset towards increased solubility, and the existence of several oxidation states for these heteroatoms expands the panel of reaction conditions applicable to trigger the extrusion process. In line with these considerations, chalcogen extrusion from heteropines has thus been explored in the frame of the “*precursor approach*” as a synthetic tool to complement retro-Diels–Alder and decarbonylation reactions and reach diversified π-conjugated polycyclic structures.

**Scheme 4:**
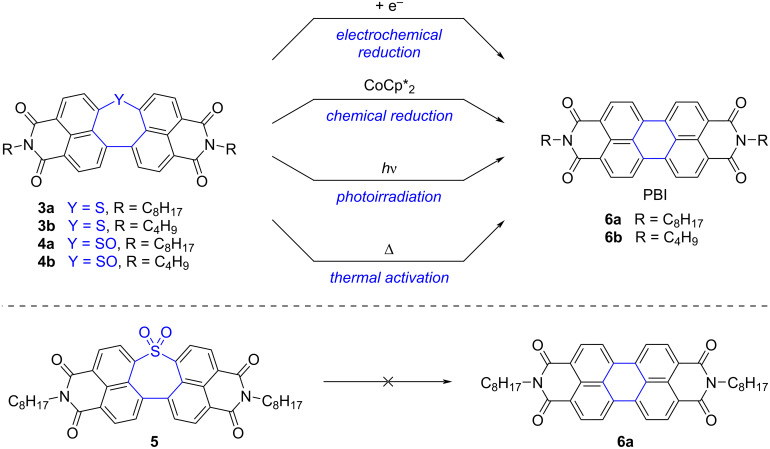
Top: Conversion of dinaphthothiepine bisimides **3a**,**b** and their sulfoxide analogues **4a**,**b** into PBIs **6a**,**b** by S-extrusion triggered by electron injection, photo- and thermal activation. Bottom: Dinaphthothiepine *S*,*S*-dioxide **5** fails to deliver PBI **6a** whatever the activation mode [57].

**Figure 1:**
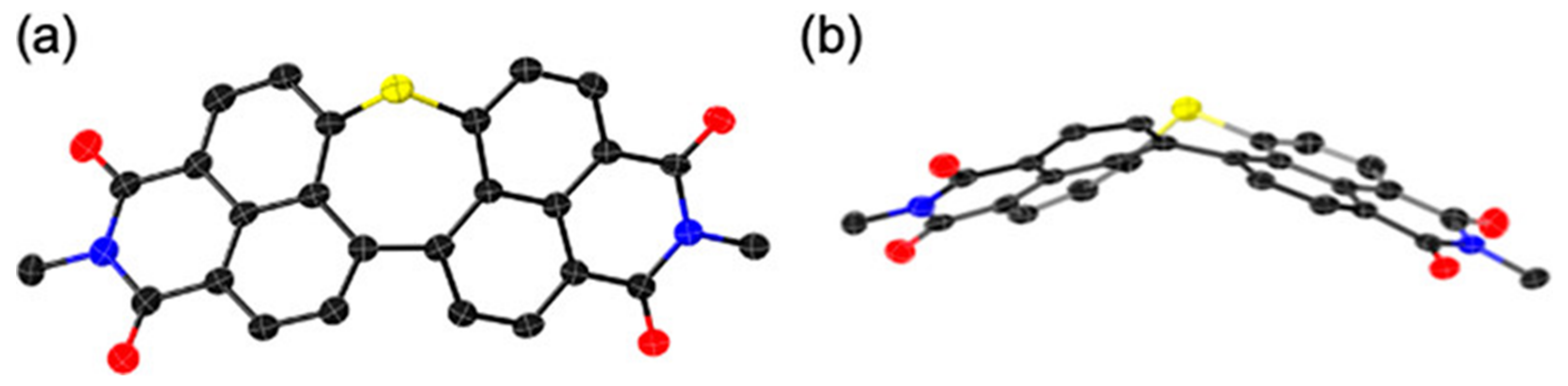
Top view (a) and side view (b) of the X-ray crystal structure of thiepine **3b** showing its bent conformation. Thermal ellipsoids are shown at the 50% probability level, and all hydrogen atoms as well as propyl groups on imide substituents have been omitted for clarity. Reprinted with permission from [57]. Copyright 2020 American Chemical Society. This content is not subject to CC BY 4.0.

In 2020, Yamada, Fukui, Shinokubo and co-workers tackled the synthesis of perylene bisimide (PBI), a valuable n-type semiconductor, via a “*precursor approach*” based on the late-stage extrusion of S-based fragments [57]. Dinaphthothiepine bisimides **3a** and **3b**, bearing octyl- and butylimide substituents respectively, were synthesized as direct soluble precursors of PBIs **6a**,**b**, along with the corresponding oxides, namely sulfoxides **4a**,**b** and sulfone **5** (Scheme 4). An X-ray crystal structure of the extended thiepine **3b** confirmed its bent structure, with a protrusion of the sulfur atom out of the π-surface (Figure 1). As expected, the non-planar thiepine derivatives **3**–**5** exhibit substantially higher solubility in organic solvents compared with the parent PBI compounds, with a 150- to 1700-fold increase in dichloromethane depending on the sulfur oxidation state. Conversion of the dinaphthothiepine core into the corresponding perylene moiety was next tested upon different stimuli, such as electron injection, thermal activation and photoirradiation. Importantly, the dinaphthothiepine *S*,*S*-dioxide, i.e., the sulfone soluble precursor **5**, failed to deliver PBI whatever the activation mode (Scheme 4, bottom). For dinaphthothiepine **3** and the related sulfoxide **4**, it was first demonstrated that electron injection is able to trigger S- (or SO-) extrusion, electrochemically but also chemically, employing decamethylcobaltocene CoCp*_2_ as a reducing agent (Scheme 4, top). Under the latter conditions, the target PBI compound **6a** was obtained in 89% yield on a ≈10 mg scale from its sulfoxide precursor **4a**. In anticipation of device fabrication, activation methods which do not require the addition of chemical reagents were next tested. Thermally-induced S-extrusion was first investigated by means of thermogravimetric analysis: the thiepine precursor **3a** exhibited a mass decrease at 300 °C in agreement with the expected elimination of sulfur to yield almost quantitatively the corresponding PBI **6a**. Its sulfoxide analogue **4a** exhibited higher thermal reactivity, with the loss of SO identified at lower temperature (225 °C). As a proof of concept, a thin-film of the latter sulfoxide precursor, spin-coated onto a glass sample, yielded full conversion to PBI **6a** in only 2 min, confirming the potential application of this precursor in electronic devices. Light irradiation was next tested as an alternative activation method, since photoextrusion of S and SO_2_ fragments are classical methods in organic synthesis [54,58–59], whereas photoextrusion of SO from bisaryl sulfoxide moieties has been evidenced more recently by Wolf et al. [60–61]. Solutions of dinaphthothiepine **3a** and its sulfoxide analogue **4a** in dichloromethane were irradiated using a high-pressure mercury lamp equipped with a sharp cut filter (λ > 380 nm), which resulted in a smooth conversion into the PBI product **6a** within 20 min. Interestingly, the sulfoxide derivative exhibited enhanced kinetics, as confirmed by UV–vis spectroscopy and NMR experiments. The latter was also reactive as a thin-film on a glass substrate, with the generation of the desired perylene upon photoirradiation with a blue LED (λ = 470 nm) [62]. Finally, cooperativity of light and heat was demonstrated on a crystalline sample of sulfoxide **4a**, where photoirradiation enhanced the SO-extrusion process at lower temperature (140 °C) than previously.

These preliminary studies thus validated the suitability of the dinaphthothiepine core and particularly its *S*-oxide as soluble precursors for perylene, with three different activation modes to generate the target PBI semiconductor. This “*precursor approach*” was next tested at the device fabrication stage in the case of OFETs. An Al_2_O_3_/SiO_2_ dielectric layer with a self-assembled monolayer of 12-cyclohexyldodecylphosphonic acid was chosen as substrate and thin-films of sulfoxide **4a** were formed by spin-coating from a chloroform solution. After thermal annealing at 230 °C for 5 min to induce SO-extrusion and subsequent vacuum deposition of the source and drain gold electrodes, the resulting OFET exhibited a typical n-type behavior with an electron mobility up to 0.41 cm^2^ V^−1^ s^−1^, comparable to vacuum deposited films of pristine PBI. When repeating the same protocol with a photoconversion of **4a** thin-film instead of thermal conversion, a decrease of the charge transport efficiency was observed, with electron mobilities of 8.4 × 10^−2^ cm^2^ V^−1^ s^−1^, which can be ascribed to the lower quality of the PBI thin-film generated upon photoextrusion due to the absence of thermal solid-state reorganization [62].

**Scheme 5:**
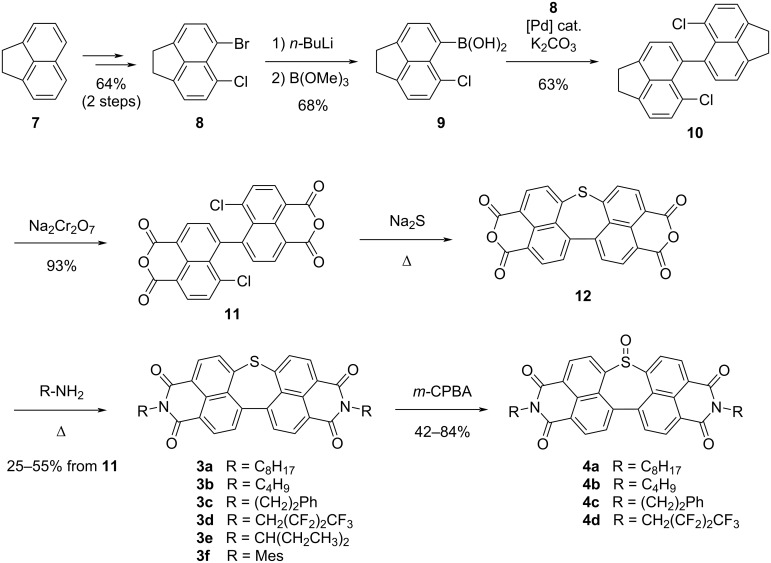
Modular synthetic route towards dinaphthothiepines **3a**–**f** and the corresponding *S*-oxides **4a**–**d**, incorporating a variety of imide substituents [62].

This work thus unequivocally revealed the potential of thiepines and thiepine *S*-oxides as direct soluble precursors of polycyclic aromatic compounds with dual stimuli-responsiveness, thus opening the way to solution-based processing for the fabrication of devices and in situ generation of the active material by late stage S-extrusion. Nevertheless, the synthetic route towards dinaphthothiepine bisimides initially reported suffered from several drawbacks: it had limited efficiency (1.2% overall yield over 8 steps for the synthesis of thiepine **3a**), it was non-scalable and displayed low modularity. Indeed, the imide groups along with their substituents were introduced at a rather early stage of the synthesis, with the ultimate synthetic step being the formation of the thiepine ring via a two-fold nucleophilic aromatic substitution by sodium sulfide (Na_2_S). Since the nature of the imide substituents is very important to control the solid state molecular arrangement, the same authors revised their initial synthetic route to allow for a late stage introduction of the imide groups, thus leading to the preparation of a series of variously-substituted soluble precursors **3a**–**f** owing to increased modularity (Scheme 5) [62]. To this aim, acenaphthene was selected as key building block and it underwent two successive regioselective halogenations to give bromochloroacenaphthene **8** in 64% overall yield. The latter was next converted into the corresponding boronic acid **9** and a Suzuki–Miyaura cross-coupling between **8** and **9** gave rise to dimer **10**, followed by the oxidation of both acenaphthene units into 1,8-naphthalic anhydrides. Installation of the thiepine ring was achieved by a double nucleophilic aromatic substitution induced by sodium sulfide (Na_2_S) to afford dinaphthothiepinetetracarboxylic anhydride **12** as key precursor of a series of thiepine bisimides bearing diverse substituents. Due to its very low solubility, the tetracarboxylic anhydride **12** was not isolated and was directly reacted with a variety of amines to yield the target soluble precursors **3a**–**f**. Diverse primary alkylamines were well tolerated, also when an electron-withdrawing polyfluoroalkyl group (**3d**) was included, and bulkier amines, such as 3-pentylamine (**3e**) or trimethylaniline (**3f**), could also be inserted. As a matter of fact, the choice of amines was limited by the temperature required for the imide formation, as high temperature (>130 °C) induced undesired thermally-activated S-extrusion as side-reaction. According to this new synthetic route, compound **3a** carrying octyl substituents was obtained on a gram-scale in 7 steps from acenaphthene, in 14% overall yield. The series of thiepines **3a**–**d** could finally be smoothly oxidized to give the corresponding sulfoxides **4a**–**d** in high yields. Thermogravimetric analysis of these thiepine *S*-oxides interestingly revealed that the temperature required for SO-extrusion is almost independent of the nature of the imide substituents, and the dinaphthothiepine *S*-oxides bearing long alkyl chains are thus very promising soluble precursors to afford n-type semiconducting layers by in situ SO-extrusion.

**Scheme 6:**
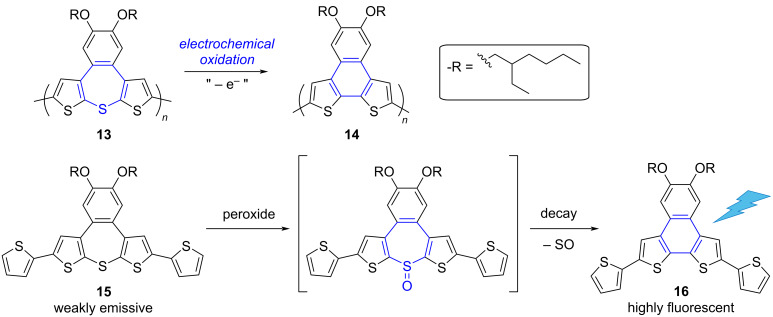
Top: Conversion of dithienobenzothiepine monomeric units into dithienonaphthalenes, upon S-extrusion triggered by electrochemical oxidation. Bottom: Exploitation of the S-extrusion process for peroxide sensing, taking advantage of the lability of oxidized dithienobenzothiepine to generate highly fluorescent dithienonaphthalene [63].

As part of their work dedicated to mechanically-responsive materials controlled by an electrochemical stimulus, Song and Swager synthesized thiepine-containing polymers designed to undergo redox-driven bent-to-planar conformational changes [63]. However, upon electrochemical oxidation of the drop-cast film of polymer **13**, the authors observed the conversion of the dithienobenzothiepine monomeric units into dithienonaphthalenes, which can be ascribed to a redox-driven S-extrusion (Scheme 6). This behavior of extended thiepines upon oxidation was further confirmed by investigating the corresponding sulfoxide, which appeared labile and readily underwent ring contraction. Since thiepine precursors (such as **15**) and the corresponding dithienonaphthalene products (e.g., **16**) exhibit very different fluorescence properties, S-extrusion from thiophene-annelated thiepines was consequently exploited for peroxide sensing.

**Scheme 7:**
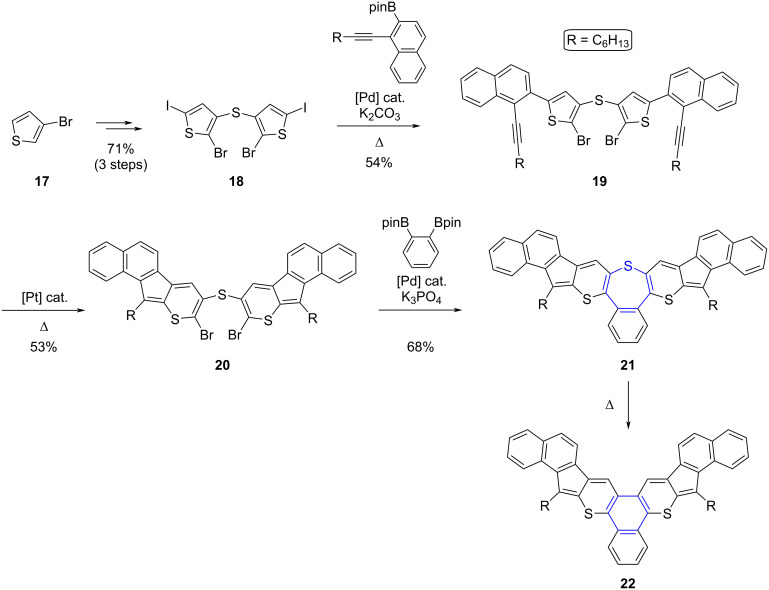
Synthesis of S-doped extended triphenylene derivative **22** from 3-bromothiophene (**17**) with the thermally-induced ring contraction of thiepine **21** as key step [64].

Very recently, Zhou et al. reported the synthesis of S-doped phenanthrene and triphenylene derivatives via the thermally-induced ring contraction of thiepine scaffolds, fused with two or three thiophene, thiopyran or polycyclic hydrocarbon moieties [64]. Indeed, a series of polyannelated thiepine derivatives were prepared according to an original approach, consisting in the introduction of the bridging sulfur atom from the outset of the synthesis and a final closure of the 7-membered ring by C–C bond formation. This is in sharp contrast with the widely adopted strategy relying on the late-stage insertion of the sulfur atom with concomitant ring closure, using either electrophilic or nucleophilic sulfur reagents. By way of example, thiepine derivative **21** was synthesized in six steps in 14% overall yield from 3-bromothiophene (**17**, Scheme 7). In the first step, the sulfur atom embedded in the final thiepine ring was introduced via a palladium-catalyzed *S*-arylation of 3-bromothiophene in the presence of potassium thioacetate, to afford the corresponding bis(thiophenyl) thioether, which then underwent successive bromination and iodination to give intermediate **18**. Next, a two-fold Suzuki–Miyaura cross-coupling occurring chemoselectively on the iodinated positions allowed the symmetric extension of the hydrocarbon scaffold, with the insertion of two naphthyl substituents carrying each an alkyne moiety on the *ortho* position. Ring expansion of both thiophene units in **19** was then triggered by platinum catalysis to generate the corresponding thiopyrans, and finally the bromine atoms located on the latter rings were exploited for the ring closure of the thiepine via a two-fold Suzuki–Miyaura cross-coupling with 1,2-phenylenediboronic pinacol ester. The resulting S-doped extended tribenzothiepine **21** proved stable under ambient conditions for several months and exhibited good solubility in common organic solvents, which is ascribed to the boat-shape conformation of the thiepine ring, thus weakening the π–π stacking intermolecular interactions. Finally, solid-state S-extrusion could be triggered in a controlled way upon thermal activation of **21**, as observed in thermogravimetric analysis, with the loss of sulfur detected at 223 °C to yield the planar S-doped triphenylene derivative **22**.

**Scheme 8:**
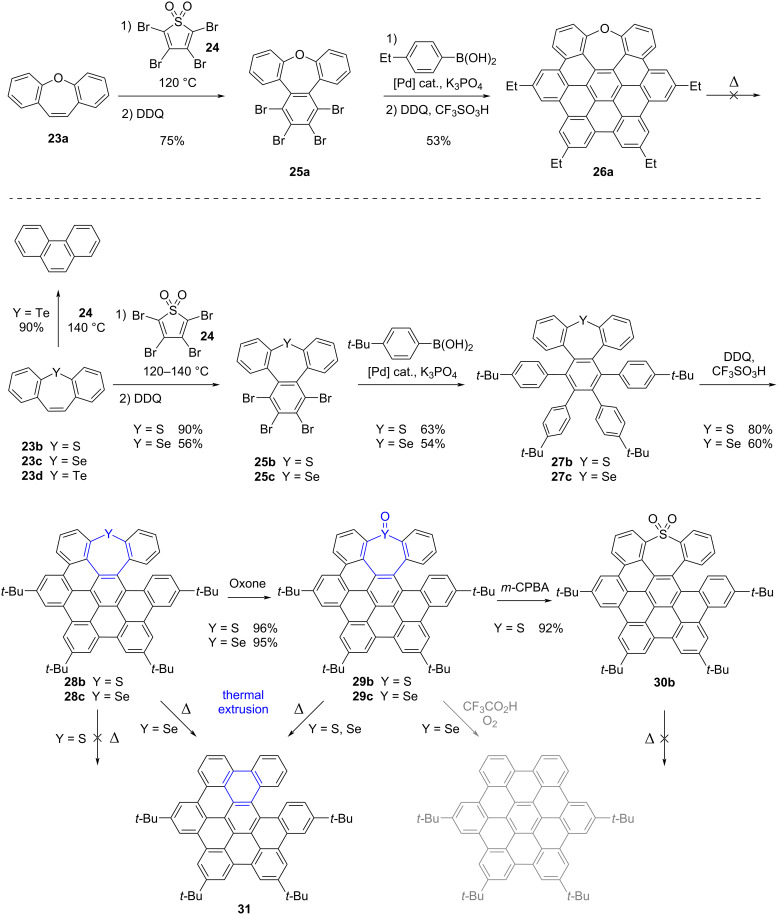
Top: Synthesis of thermally-stable O-doped HBC **26a**. Bottom: Synthesis of S- and Se-based soluble precursors of *seco*-HBC **31** and their conversion by chalcogen extrusion upon thermal activation [65].

In the same year, An et al. reported their investigations regarding chalcogen-doped hexa-*peri*-benzocoronene (HBC) derivatives and their controllable conversion into hydrocarbon nanographenes by ring contraction of the heteropine moiety [65]. The authors first tackled the synthesis of a series of HBC precursors incorporating a group-16 heteroatom embedded in a seven-membered ring. In the case of oxygen, the fully cyclized HBC **26a** containing an oxepine ring and exhibiting a saddle-shape tridimensional structure was successfully prepared (Scheme 8, top). Conversely, with heavier chalcogens, only partially cyclized backbones, namely *seco*-HBCs **28**, were obtained (Scheme 8, bottom). The synthesis of these helical compounds was based on S-, Se- or Te-dibenzo[*b*,*f*]heteropines **23b**–**d** as key intermediates, which were engaged in a Diels–Alder reaction with tetrabromothiophene *S*,*S*-dioxide **24** at 120–140 °C to yield the corresponding *S*- and *Se*-tribenzo[*b*,*d*,*f*]heteropines, **25b** and **25c** respectively, after oxidative aromatization mediated by DDQ. In the case of tellurepine **23d**, attempts of thermally-activated Diels–Alder reaction resulted in Te-extrusion to afford phenanthrene as the main product. This result highlights the higher sensitivity of the Te-heteropines compared to S- and Se-counterparts towards thermal chalcogen extrusion. Next, four-fold Suzuki–Miyaura coupling of the tetrabrominated tribenzoheteropines **25b** and **25c** delivered compounds **27b** and **27c** as direct precursors of S- and Se-inserted HBCs. The subsequent Scholl reaction allowed the formation of four new C–C bonds, but failed to afford the fully cyclized chalcogen-embedded HBC whatever the conditions tested, presumably due to large strain in line with the size of S and Se atoms as compared with O. The helical S- and Se-doped *seco*-HBCs **28b** and **28c** were thus obtained as racemic mixtures in three steps from dibenzoheteropines **23b** and **23c** in 45% and 18% overall yield, respectively. To evaluate the impact of the heteroatom oxidation state on reactivity, the sulfur and selenium oxides were next prepared via selective oxidation of thiepine and selenepine to give rise to the corresponding sulfoxide **29b**, selenoxide **29c** and sulfone **30b** in excellent yields.

All the prepared chalcogen-doped HBC derivatives exhibit good solubility in organic solvents, in line with their highly distorted saddle-shaped or helical structure induced by the embedded heteropine ring. By way of example, the solubility of SeO-doped *seco*-HBC **29c** is more than 100-fold higher than pristine HBC carrying four *tert*-butyl groups in both dichloromethane and ethanol.

Ring contraction was next attempted on the series of heteropines synthesized previously. Importantly, photoirradiation at 254 nm or 365 nm failed to induce any chalcogen extrusion. When thermal activation was tested on solid samples, oxepine **26a**, thiepine **28b** and thiepine *S*,*S*-dioxide **30b** were not converted upon heating at 250 °C for 2 hours, thus highlighting their high thermal stability. Conversely, selenepine **28c** and selenepine *Se*-oxide **29c** were quantitatively converted into the *seco*-HBC **31** by thermal activation at 200 °C for 5 min, as evidenced by UV–visible absorption and HPLC monitoring. SO-extrusion from thiepine *S*-oxide **29b** was also successfully triggered in the solid state, but it required prolonged heating at higher temperature (250 °C). In this work, *seco*-HBC **31** was thus successfully obtained via thermally-induced ring contraction of certain heteropines with the concomitant cheletropic elimination of chalcogen fragments. These results nicely illustrate how the nature, as well as the oxidation state of the embedded chalcogen atom, allows controlling the stability of the heteropine, and thus its propensity to undergo thermal extrusion. Although beyond the scope of this review, it is noteworthy that the authors successfully converted the selenepine *Se*-oxide **29c** into the corresponding planar HBC at room temperature upon acidic treatment under air, thus triggering SeO-elimination as well as cyclodehydrogenation in a single step.

As mentioned above, oxepine derivatives display higher stability compared to other chalcogen heteropines and direct oxygen extrusion from oxepines under photo- or thermal activation has not been reported. Oxygen thus appears as the chalcogen element of choice to obtain stable heteropines embedded in π-CPCs and study the properties of such non-planar compounds.

**Scheme 9:**
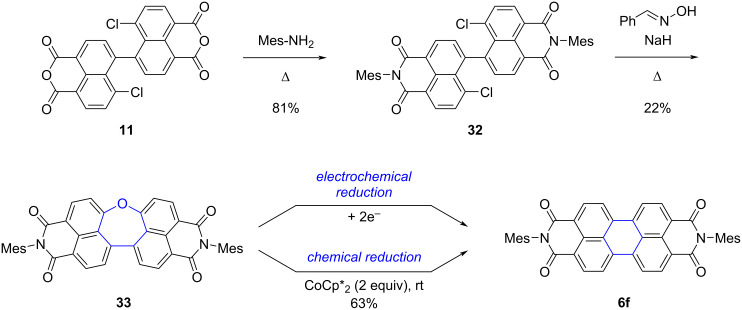
Synthesis of dinaphthooxepine bisimide **33** and conversion into PBI **6f** by O-extrusion triggered by electron injection [66].

Very recently, Fukui, Shinokubo and co-worker reported the synthesis of dinaphthooxepine bisimides [66], which are analogues of the dinaphthothiepine bisimides presented above with an oxygen atom substituting the sulfur atom (Scheme 9). Their synthesis was successfully achieved in two steps from the known intermediate 5,5’-linked 4-chloro-1,8-naphthalic anhydride dimer **11**, already exploited in the previous thiepine synthesis (see Scheme 5). Installation of the imide groups was performed first and dianhydride **11** was thus treated with 2,4,6-trimethylaniline to afford the corresponding bisimide **32** in good yield. Subsequently, reaction with α-benzaldoxime in the presence of sodium hydride resulted in the formation of the oxepine ring by a double substitution reaction, to yield the desired dinaphthooxepine **33**.

**Figure 2:**
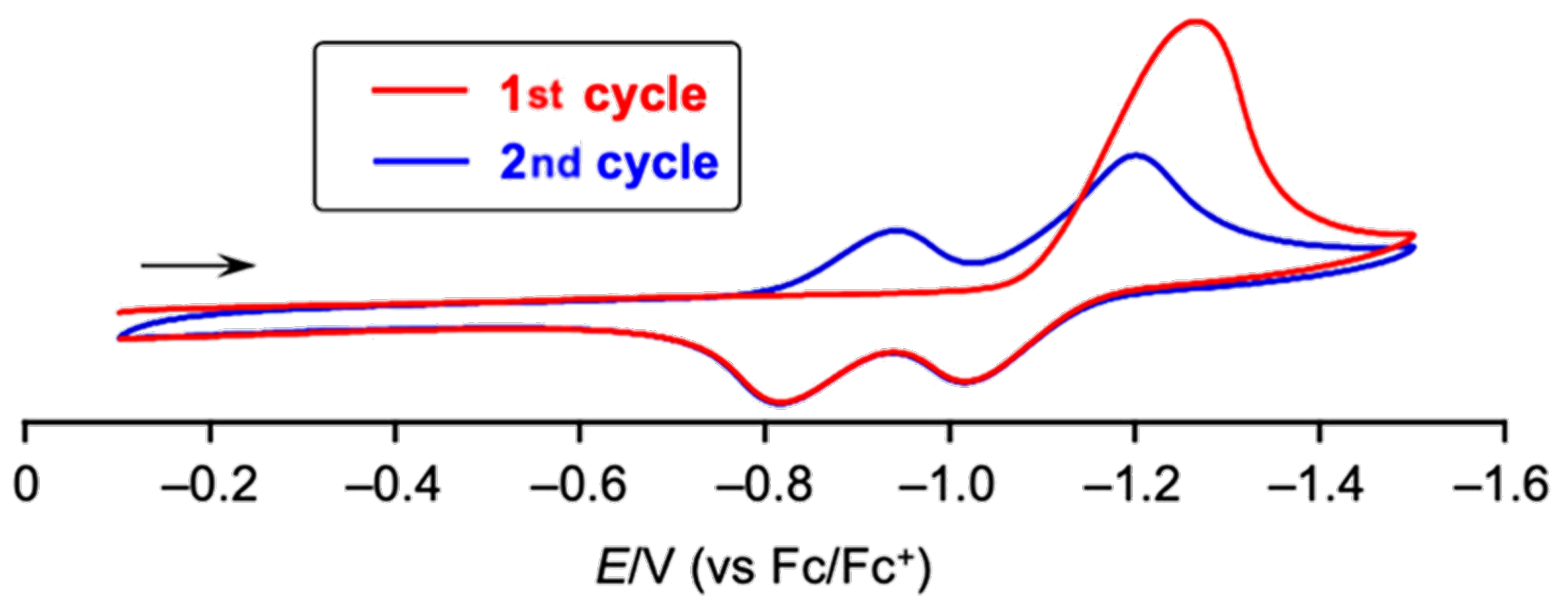
Cyclic voltammogram of dinaphthooxepine **33**, evidencing the irreversibility of the reduction process during the first cycle, leading to the formation of PBI **6f** upon redox-triggered O-extrusion. Reprinted with permission from [66]. Copyright 2023 American Chemical Society. This content is not subject to CC BY 4.0.

The non-planar character of dinaphthooxepine bisimides was confirmed by X-ray crystal structure, and stability towards thermal or photoactivation was also established. Cyclic voltammetry studies revealed that reduction of dinaphthooxepine **33** proceeds irreversibly, giving rise to a new chemical species (Figure 2), and this was further confirmed by chemical reduction in the presence of decamethylcobaltocene CoCp*_2_ at room temperature. Using two equivalents of this 1-electron reducing agent, O-extrusion takes place with concomitant ring contraction to give the corresponding PBI **6f**, isolated in 63% yield after oxidative quenching and work-up. Contrary to photo- or thermal activation, it is important to note that the mechanism for O-extrusion involves in this case the dianion of dinaphthooxanorcaradiene bisimide valence isomer obtained upon electron injection and not its neutral form, as shown by UV–vis absorption and DFT studies. The mechanism of the final release of oxygen still remains unclear.

This example shows that a careful molecular design allows endowing chalcogen heteropines with thermal- and photostability by the choice of oxygen as heteroatom, while keeping the possibility to trigger chalcogen extrusion via a radically different mechanistic route, as a result of the injection of electrons. Here, the presence of the imide functions appended to the naphthyl scaffold is crucial, since previously reported bare and aryl-substituted dinaphthooxepines exhibited reversible reduction [67].

In summary, owing to the heteroatom-dependent behavior of heteropines, it is possible to control the structure of the target compound: on the one hand, thermally stable chalcogen-doped π-CPCs with distorted structures can be obtained and their intrinsic properties investigated, and on the other hand, with a subtle choice of the chalcogen element and its oxidation state, controlled extrusion coupled to 7-membered ring contraction will lead to planar benzenoid π-CPCs in situ, in response to diverse stimuli.

In the next section, chalcogen extrusion resulting from the contraction of 6-membered rings upon photo- and thermal activation will be detailed.

### Contraction of 6-membered rings coupled to chalcogen extrusion

**Scheme 10:**
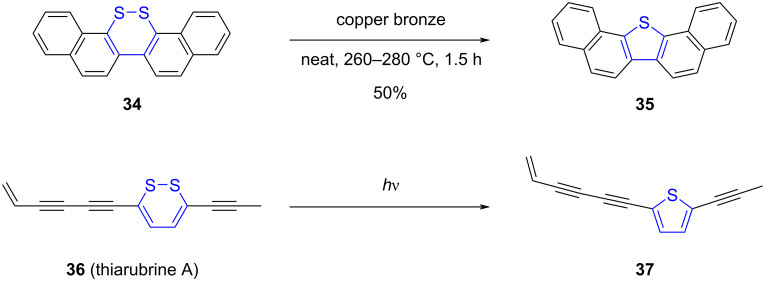
Top: Early example of 6-membered ring contraction with concomitant S-extrusion leading to dinaphthothiophene [69]. Bottom: Photoactivated S-extrusion occurring in natural product thiarubrine A [70].

Ring contraction of 6-membered heterocycles containing chalcogen atoms has been employed as a strategy to synthesize π-CPCs containing 5-membered rings. The controlled extrusion of sulfur from 1,2-dithia-3,5-cyclohexadienes (1,2-dithiins) was exploited as early as in the 1950s to prepare extended thiophene derivatives, such as dibenzo- and dinaphthothiophene (Scheme 10, top) [68–69]. Similarly to the early transformations of thiepines, sulfur elimination was triggered by copper bronze at high temperature, thus leading to the conversion of the 6-membered cyclic disulfide into the corresponding thiophene.

The 1,2-dithiin scaffold is found in natural products such as thiarubrines A and B, which have been shown to be particularly sensitive to light [70]. Upon exposure to UV or visible light, thiarubrine A (**36**) undergoes a desulfurization process to give rise to the corresponding thiophene **37**, exhibiting enhanced antibiotic activity (Scheme 10, bottom). This naturally-occurring S-extrusion under light activation thus prompted the investigation of 1,2-dithia-3,5-cyclohexadienes as synthetic photoprecursors of thiophene-based π-CPCs.

**Scheme 11:**
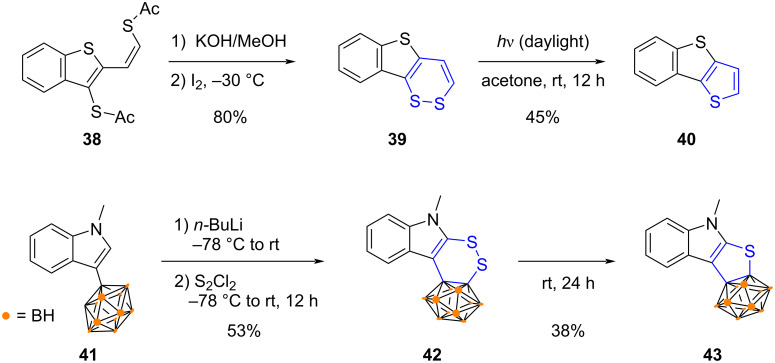
Examples of S-extrusion from annelated 1,2-dithiins under photoactivation (top) or thermal activation (bottom) [71–72].

In this sense, Schroth et al. reported the synthesis of thienothiophene and benzothienothiophene **40** from dithiin key intermediates (Scheme 11, top) [71]. Benzo[4,5]thieno[3,2-*c*][1,2]dithiin (**39**) was prepared by intramolecular oxidative coupling of a dithiolate generated in situ from dithioacetate **38**, and subsequent exposition to daylight of a solution of **39** triggered ring contraction with concomitant elimination of sulfur to yield target benzothienothiophene **40**. Importantly, light-induced S-extrusion from 1,2-dithiins appears to be structure-dependent, as di-annelated dithieno[1,2]dithiin analogues (i.e., 1,2-dithiins fused with two thiophene moieties) proved stable upon light exposure.

In their work aiming at deciphering the electronic interactions between carboranes and chalcogen-doped π-conjugated heterocycles, Yang and co-workers devised a two-step strategy for the synthesis of carborane-fused thiophene **43** (Scheme 11, bottom) [72]. Double lithiation of carboranyl indole **41** was followed by trapping with disulfur dichloride as chalcogen source to afford 1,2-dithiin **42**. At room temperature, the latter then spontaneously evolved via a S-extrusion to yield the desired carborane-fused thiophene **43**. The same method was further employed to prepare other analogues incorporating a group-16 heteroatom.

The strategy involving 6-membered ring contraction by chalcogen extrusion thus appears as highly promising for the synthesis of conjugated materials containing thiophene or selenophene moieties. However, 1,2-dichalcogenin cycles stabilized in highly conjugated scaffolds are not easily converted by light or thermal activation, as mentioned above, and their reactivity in the absence of external reagent (i.e., their photo- and thermal reactivity) has been far less explored than for heteropines. Nowadays, the most common protocol to trigger extrusion from 1,2-dichalcogenins and generate S- and Se-doped π-CPCs still relies on copper (as nanopowder) at high temperature under neat conditions [73–77].

**Scheme 12:**
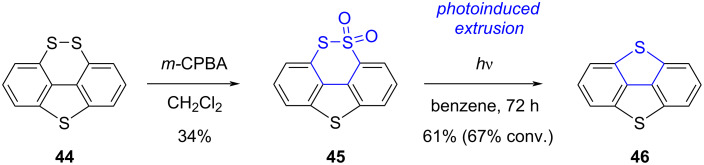
Synthesis of dibenzo[1,4]dithiapentalene upon photoextrusion of SO_2_ [78].

In order to promote light-induced ring contraction of 1,2-dithiin scaffolds, Furukuwa et al. explored the reactivity of precursors bearing one sulfur atom in a higher oxidation state [78]. In their synthesis of dibenzo[1,4]dithiapentalene **46**, the 1,2-dithiin precursor **44** was first oxidized by means of *m-*CPBA into thiosulfonate **45**, which was subsequently irradiated with light in benzene to give dithiapentalene **46** in 61% yield upon SO_2_-extrusion (Scheme 12).

**Scheme 13:**
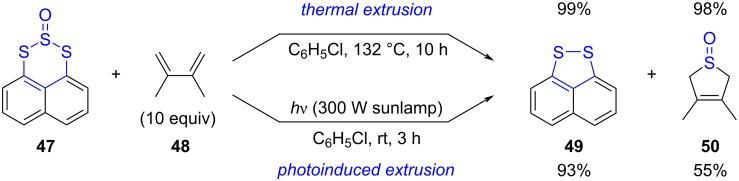
Extrusion of SO in naphthotrithiin-2-oxides for the synthesis of 2,5-dihydrothiophene 1-oxides [79].

In the same context, the thermal and photochemical extrusion of SO has been exploited as well in 6-membered rings containing a sulfoxide moiety. For instance, Grainger and co-workers designed a series of naphthotrithiin-2-oxides, such as **47**, to act as SO transfer reagents (Scheme 13) [79]. These triplet SO precursors were prepared by treatment of 1,8-naphthalene dithiols with thionyl chloride to generate the trisulfide-2-oxide moiety. Thermal activation of **47** in refluxing chlorobenzene triggered the elimination of SO, which was subsequently trapped by a variety of conjugated dienes such as 2,3-dimethylbutadiene (**48**), to afford in good to excellent yields 2,5-dihydrothiophene *S*-oxides, along with the 5-membered 1,8-naphthalene disulfide **49** resulting from the ring contraction of precursor **47**. Such SO transfer process, favoured by the *peri*-interaction existing in the trisulfide-2-oxide substrate, was also successfully promoted by light and the cyclic sulfoxide **50** was obtained under milder conditions in 55% yield. For increased synthetic utility, the latter could be converted in a straightforward manner into the corresponding thiophene under Pummerer conditions.

**Scheme 14:**
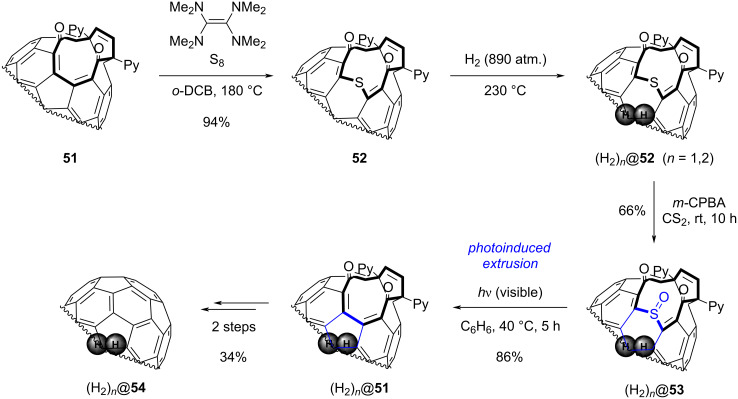
SO-extrusion as a key step in the synthesis of fullerenes (C_60_ and C_70_) encapsulating H_2_ molecules [80,82].

A 6-membered ring contraction through SO-extrusion was also introduced as key step in a molecular “surgical” method developed by Murata, Komatsu et al. [80]. In this approach, C_60_ or C_70_ fullerenes are modified stepwise towards an open-cage derivative in order to encapsulate small molecules such as H_2_ [81–82]. Eventually, the chemical modifications are reversed to close the hole and give an endohedral fullerene. As depicted in Scheme 14, the 12-membered ring orifice of fullerene **51** was first enlarged by electrophilic addition of sulfur. After encapsulation of H_2_ molecule(s) (*n* = 1 or 2), four synthetic steps were required to reduce the hole size and ultimately obtain cage-closed compound (H_2_)*_n_*@**54**. In order to remove sulfur and regenerate the initial 5-membered carbocycle, the thiopyran scaffold in (H_2_)*_n_*@**52** was first oxidized with *m-*CPBA to yield the corresponding sulfoxide (H_2_)*_n_*@**53**. As a key step, the latter was next subjected to photoinduced SO-extrusion under visible light, leading to a ring contraction in 86% yield. At that stage, the initial ring size of precursor **51** was recovered, and two further steps including a McMurry reaction and a thermolysis were then necessary to obtain endohedral fullerene (H_2_)*_n_*@**54**.

As seen in the two first parts of this review, synthetic strategies involving a late-stage ring contraction, accompanied with the extrusion of a chalcogen fragment, have led to the preparation of a variety of π-CPCs, displaying a wide range of 2D or 3D structures. While contraction of 6-membered rings has not been exploited yet for the in situ generation of molecular materials, chalcogen extrusion from group-16 heteropines was successfully implemented as a key step in the fabrication of devices with highly promising properties. Elimination of chalcogen fragments coupled to ring contraction thus appears as a powerful synthetic tool to complement retro-Diels–Alder reactions and decarbonylations widely used in the frame of the “*precursor approach*”, and further contributions to this field will most probably widely expand the range of π-conjugated polycyclic structures to be investigated as active materials in opto-electronic devices.

In contrast with the two previous sections, the last part of this review focuses on extrusion of chalcogen fragments which does not involve ring contraction. In such case, the soluble precursors display a bridged structure, reminiscent of the bicyclo[2.2.2]octadiene, bicyclo[2.2.2]octane-2,3-dione and norbornadien-7-one exploited as photo- and thermal precursors of acenes, as described in the introduction.

### Chalcogen extrusion from bridged precursors: access to acenes

Deoxygenation of endoxides belongs to the classical methods used in organic chemistry to generate polycylic aromatic hydrocarbons, through a reductive aromatization relying on a variety of chemical reagents [4]. The non-planar character of the bridging epoxide moieties endows the precursors with increased solubility, and endoxides have thus been envisioned as soluble precursors of acenes. Since the addition of chemical reagents is not desirable in this context, thermal activation and electron injection were investigated as stimuli to trigger O-extrusion.

**Scheme 15:**
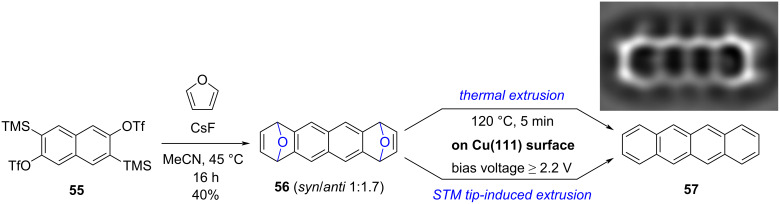
Synthesis of diepoxytetracene precursor **56** and its on-surface conversion into tetracene upon O-extrusion. Inset in the top right shows a Laplace-filtered AFM image of a tetracene molecule produced on a Cu(111) surface. Inset adapted with permission from [83]. Copyright 2016 American Chemical Society. This content is not subject to CC BY 4.0.

Moresco, Peña and co-workers thus designed diepoxytetracene precursor **56**, displaying an oxabicyclo moiety at each extremity (Scheme 15) [83]. The latter was prepared in one step via a double Diels–Alder reaction between furan and 2,6-naphthodiyne, generated in situ from bistriflate **55** in the presence of cesium fluoride. Diepoxide **56** was obtained in 40% yield as a *syn*/*anti* mixture in a 1:1.7 ratio.

Diepoxide **56** (as a diastereoisomeric mixture) was deposited on a Cu(111) surface by sublimation under UHV conditions and the molecular species adsorbed on the metallic surface were characterized by combined STM and nc-AFM at low temperature (5 K), thus evidencing the presence of the two bridging oxygens. Next, the STM tip-induced extrusion of oxygen was investigated: application of a bias voltage of ≈2.2 V on top of one 1,4-epoxide bridge led to a planarization of the carbon-based scaffold along with the elimination of one oxygen atom to give a monoepoxide species. When the latter was submitted to a second voltage pulse, a fully planar and aromatic backbone made of four linearly-fused benzene rings was observed on STM and AFM images, thus unambiguously proving the formation of tetracene **57**. Importantly, the oxygen atoms extruded from precursor **56** were observed on the copper surface in close proximity to the converted molecule.

In contrast with this tip-induced conversion of soluble precursor **56**, which occurs stepwise and gives rise to one single target molecule after each manipulation cycle, thermal activation of the population of adsorbed diepoxides **56** was next attempted. Annealing at 120 °C for 5 min successfully promoted the O-extrusion to yield the corresponding tetracene molecules, without any trace of partially-converted monoepoxide intermediate.

**Scheme 16:**
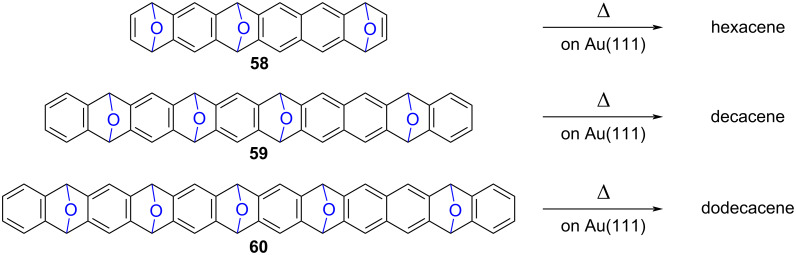
Soluble precursors of hexacene, decacene and dodecacene incorporating 1,4-epoxides in their hydrocarbon scaffold, and on-surface conversion into higher acenes by thermally-activated O-extrusion [84–86].

As mentioned in the introduction section, the quest for longer unsubstituted acenes has captivated chemists and physicists for almost two decades now, with the aim to explore their electronic properties. In this context, on-surface synthesis of higher acenes according to the “*precursor approach”* is particularly interesting, since the as-prepared π-extended structures can undergo in-depth structural characterization and electronic investigations in situ at the single molecule scale, thanks to scanning probe microscopy and spectroscopy. The synthetic route relying on O-extrusion was thus transposed to the synthesis of hexacene [84], decacene [85] and ultimately dodecacene [86], which is the longest acene prepared to date. The related soluble precursors **58**–**60** incorporate 3 to 5 oxabicyclo moieties in their hydrocarbon scaffold (Scheme 16), and are sufficiently stable and soluble to undergo purification by column chromatography prior to their on-surface deposition.

**Scheme 17:**
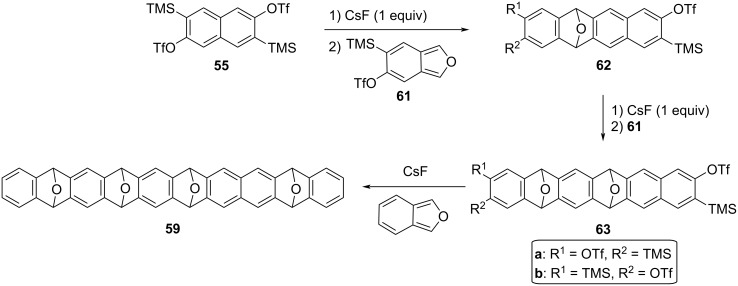
Synthesis of tetraepoxide **59** as soluble precursor of decacene [85].

By way of example, the synthesis of decacene precursor **59** was achieved in three synthetic steps by iterative [4 + 2] cycloadditions with an overall yield of 4.7% (Scheme 17). The synthesis of epoxyacene **59** started with the mono-Diels–Alder reaction of naphthodiyne precursor **55** with isobenzofuran **61** to give monoepoxide **62** as a mixture of regioisomers. Further treatment of the latter with one equivalent of cesium fluoride allowed the regioselective Diels–Alder with the previous diene **61**, to afford **63** as another mixture of position isomers. Finally, reaction of this bisaryne precursor with an excess cesium fluoride and bare isobenzofuran gave rise to the end-capped soluble precursor **59** via a double cycloaddition process.

**Figure 3:**
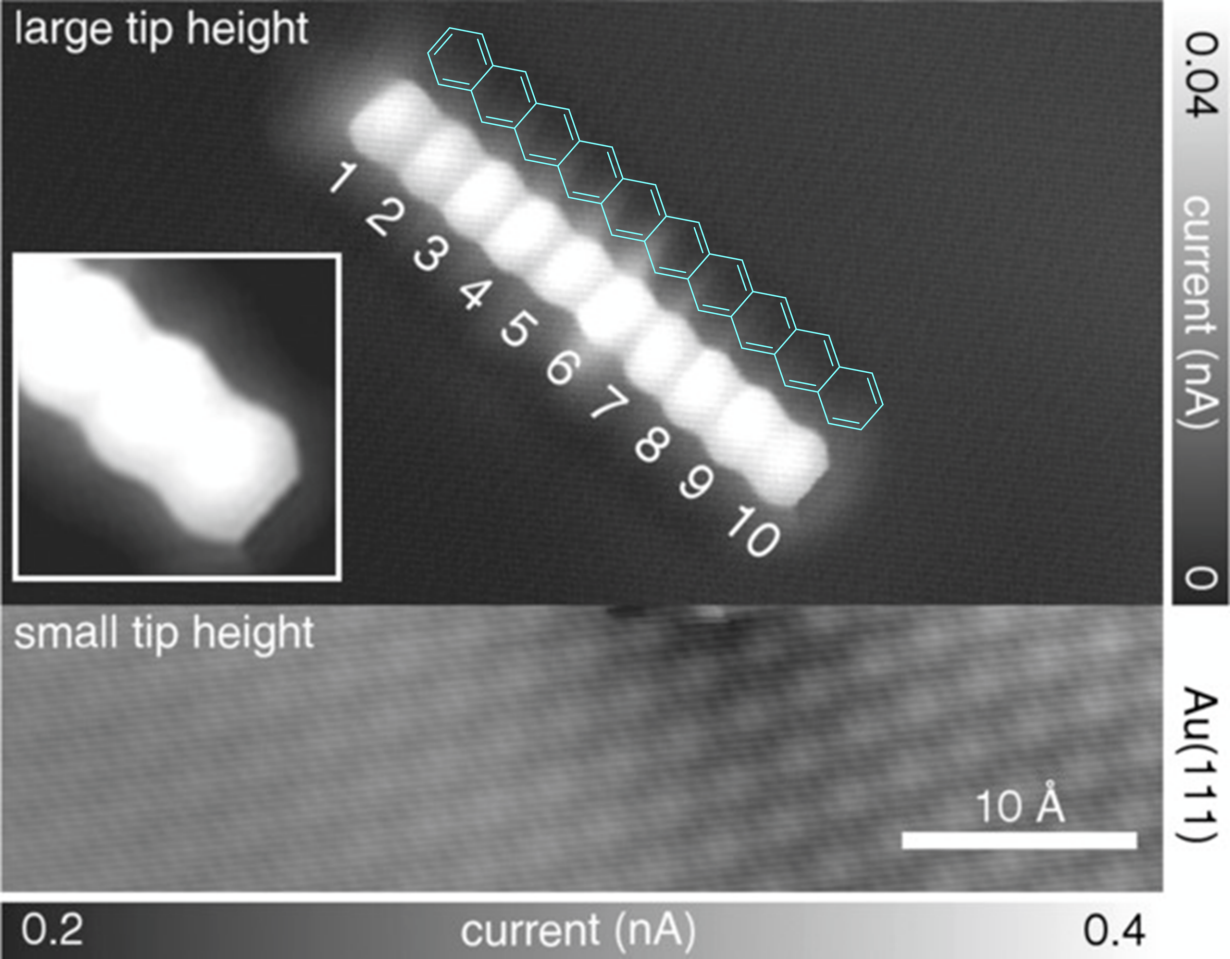
Constant-height STM measurement of decacene on Au(111) using a CO-functionalized tip (sample voltage *V* = 20 mV), with the chemical structure of decacene superimposed in blue as a guide to the eye. Two different tip height domains were employed to resolve the Au(111) lattice as well as the adsorbed molecule with atomic resolution. Adapted with permission from [85], J. Krüger et al., “Decacene: On-Surface Generation”, *Angew. Chem., Int. Ed.*, with permission from John Wiley and Sons. Copyright © 2017 Wiley-VCH Verlag GmbH & Co. KGaA, Weinheim. This content is not subject to CC BY 4.0.

All polyepoxides **58**–**60** were deposited by sublimation on a Au(111) surface, triggering in some cases partial deoxygenation during this physical vapor deposition. Subsequent annealing in the 150–220 °C range led to the thermally-induced surface-assisted cleavage of oxygen to give rise to the corresponding acenes, namely hexacene, decacene and dodecacene. Their structure was unambiguously assigned thanks to high-resolution STM and AFM images, revealing the linear fusion of benzene rings with a zigzag edge (Figure 3), and their electronic properties were investigated at the single-molecule scale, with a particular attention devoted to the evolution of the HOMO–LUMO gap along the incremental series of acenes.

## Conclusion

Over the last decades, the “*precursor approach*” has proved particularly valuable for the preparation of insoluble and unstable π-CPCs, which cannot be synthesized via in-solution organic chemistry, for their improved processing, as well as for their structural characterization and electronic investigation both at the material and single-molecule scales. Beside reactions involving the elimination of carbon-based small molecules, i.e. retro-Diels–Alder and decarbonylation processes, the late-stage extrusion of chalcogen fragments has emerged as a highly promising synthetic tool to access a wider variety of π-conjugated polycyclic structures upon thermal activation, photoirradiation and redox control.

While contraction of 6-membered rings has not been exploited yet for the in situ generation of molecular materials, chalcogen extrusion from group-16 heteropines was successfully implemented as a key step in the fabrication of devices. Notably, the use of heteropines as soluble precursors allowed to expand the “*precursor approach*” to new families of organic semiconductors, such as PBIs and triphenylenes. It is important to mention here that, depending on the nature of the soluble heteropine precursor, the extrusion process may lead to volatile and/or non-volatile chalcogen species. In the latter case, the target π-CPCs may be contaminated by inorganic impurities, especially when extrusion reactions are performed in the solid state, which may in turn alter the properties of the resulting molecular materials. Further investigations regarding the fate of the extruded chalcogen fragments and their impact on device performances are thus desirable and will surely drive the design of future chalcogen-based soluble precursors.

Chalcogen extrusion has also been investigated in the absence of concomitant ring contraction, and in this context, endoxides have been envisioned as soluble precursors of higher acenes. Conversion of polyepoxide precursors was successfully achieved on metallic surfaces, at the single-molecule scale, upon STM tip-induced or thermally-activated O-extrusion to yield acenes of increasing lengths, up to the long-sought dodecacene.

Following the pioneering works detailed in this review, further contributions to this field will most probably widely expand the range of π-conjugated polycyclic structures accessible via chalcogen extrusion in the upcoming years.

## Data Availability

Data sharing is not applicable as no new data was generated or analyzed in this study.
